# Slow Conduction in the Border Zones of Patchy Fibrosis Stabilizes the Drivers for Atrial Fibrillation: Insights from Multi-Scale Human Atrial Modeling

**DOI:** 10.3389/fphys.2016.00474

**Published:** 2016-10-25

**Authors:** Ross Morgan, Michael A. Colman, Henry Chubb, Gunnar Seemann, Oleg V. Aslanidi

**Affiliations:** ^1^Division of Imaging Sciences and Biomedical Engineering, Department of Biomedical Engineering, King's College LondonLondon, UK; ^2^School of Biomedical Sciences, University of LeedsLeeds, UK; ^3^Institute for Experimental Cardiovascular Medicine, University Heart Center - Bad Krozingen, Medical Center - University of FreiburgFreiburg, Germany

**Keywords:** atrial fibrillation, fibrosis, modeling and simulation, arrhythmia mechanisms, MR imaging

## Abstract

**Introduction:** The genesis of atrial fibrillation (AF) and success of AF ablation therapy have been strongly linked with atrial fibrosis. Increasing evidence suggests that patient-specific distributions of fibrosis may determine the locations of electrical drivers (rotors) sustaining AF, but the underlying mechanisms are incompletely understood. This study aims to elucidate a missing mechanistic link between patient-specific fibrosis distributions and AF drivers.

**Methods:** 3D atrial models integrated human atrial geometry, rule-based fiber orientation, region-specific electrophysiology, and AF-induced ionic remodeling. A novel detailed model for an atrial fibroblast was developed, and effects of myocyte-fibroblast (M-F) coupling were explored at single-cell, 1D tissue and 3D atria levels. Left atrial LGE MRI datasets from 3 chronic AF patients were segmented to provide the patient-specific distributions of fibrosis. The data was non-linearly registered and mapped to the 3D atria model. Six distinctive fibrosis levels (0–healthy tissue, 5–dense fibrosis) were identified based on LGE MRI intensity and modeled as progressively increasing M-F coupling and decreasing atrial tissue coupling. Uniform 3D atrial model with diffuse (level 2) fibrosis was considered for comparison.

**Results:** In single cells and tissue, the largest effect of atrial M-F coupling was on the myocyte resting membrane potential, leading to partial inactivation of sodium current and reduction of conduction velocity (CV). In the 3D atria, further to the M-F coupling, effects of fibrosis on tissue coupling greatly reduce atrial CV. AF was initiated by fast pacing in each 3D model with either uniform or patient-specific fibrosis. High variation in fibrosis distributions between the models resulted in varying complexity of AF, with several drivers emerging. In the diffuse fibrosis models, waves randomly meandered through the atria, whereas in each the patient-specific models, rotors stabilized in fibrotic regions. The rotors propagated slowly around the border zones of patchy fibrosis (levels 3–4), failing to spread into inner areas of dense fibrosis.

**Conclusion:** Rotors stabilize in the border zones of patchy fibrosis in 3D atria, where slow conduction enable the development of circuits within relatively small regions. Our results can provide a mechanistic explanation for the clinical efficacy of ablation around fibrotic regions.

## Introduction

Atrial fibrillation (AF) is the most common sustained cardiac arrhythmia, affecting 6 million people in Europe alone and imposing a huge healthcare burden on the society. Despite well-developed clinical guidelines for the management of AF, anti-arrhythmic therapy success rates remain suboptimal due to complexity of the disease and lack of knowledge of its mechanisms (Calkins et al., 2012). Catheter ablation is a standard clinical treatment aimed at eliminating the arrhythmogenic electrical drivers in the atria by destroying the underlying tissue substrate with a localized energy delivery. However, precise driver locations in a patient are generally unknown, and the long-term success rates of empirical ablation procedures (such as the pulmonary vein isolation) are low, with the disease recurring in 30–50% of patients (Schotten et al., 2011; Calkins et al., 2012).

Both the genesis of AF and success of ablation therapy have been strongly linked with atrial fibrosis (Marrouche et al., 2014; Gal and Marrouche, 2015; Kottkamp et al., 2015). Fibrosis levels are higher in AF patients compared to healthy subjects and correlate positively with AF recurrence (Marrouche et al., 2014) after ablation. Moreover, ablation around fibrotic areas identified from late-gadolinium enhanced (LGE) MRI (Gal and Marrouche, 2015) or electro-anatomical mapping (Kottkamp et al., 2015) has been shown to improve therapy success rates. Mechanistically, structural and functional tissue heterogeneities associated with fibrosis have generally been linked to arrhythmogenesis, for example in ventricular infarction border zones (BZ) (Rutherford et al., 2012). Such border heterogeneities can be presented as large collagen deposits within the functional myocardium, which can slow down or block the propagation of electrical excitation waves, creating conditions for the generation and sustenance of re-entrant drivers (rotors).

Recent studies (Jadidi et al., 2013; Haissaguerre et al., 2016) have demonstrated that areas adjacent to dense fibrosis in the atria have also shown high levels of arrhythmogenic activity. These myocardial areas, whilst still fibrotic, are not damaged sufficiently to fully inhibit wave propagation and have a high correlation to complex high-frequency electrical activity (Jadidi et al., 2013). Structurally and functionally these regions are reminiscent of ventricular infarction BZs, which commonly surround dense fibrotic areas (such as scar) and have also been shown to exhibit low levels of electrical activity. This similarity suggests that patient-specific distributions of atrial fibrosis can also determine the dynamics of electrical drivers sustaining AF. A recent computational study has shown that AF can be perpetuated by rotors persisting in fibrotic BZs with specific statistical characteristics (Zahid et al., 2016). However, mechanistic links between atrial fibrosis and the genesis of AF drivers are unclear.

Computational modeling provides a powerful tool for integrating multi-scale atrial data and dissecting the mechanisms of arrhythmia and anti-arrhythmic treatments *in-silico*, (Colman et al., 2013; McDowell et al., 2015; Zahid et al., 2016) which is extremely challenging in a purely experimental or clinical setting. Recent computational studies have shown the importance of heterogeneities associated with fibrosis, (McDowell et al., 2015; Zahid et al., 2016) as well as other factors, (Aslanidi et al., 2011; Colman et al., 2013) in the generation and sustenance of AF. However, even the most advanced models have only considered step-wise heterogeneities, such as discontinuous transitions from healthy or fibrotic tissue with no genuine transitional zone (Zahid et al., 2016)—and hence, have not explored the mechanistic effects of patchy fibrotic BZs. Besides, electrophysiological models developed for ventricular (rather than atrial) fibroblasts have commonly been utilized (McDowell et al., 2015).

The current study aims to develop both a novel model for atrial fibroblast, and a novel image-based approach to modeling gradual transitions in properties between healthy and fibrotic atrial tissue. These novel electrophysiological and structural data will be integrated into multi-scale biophysical models of the 3D human atria. The detailed models will then be applied to substantiate mechanistic links between patient-specific distributions of fibrosis and location of drivers sustaining AF. Specifically, we will explore a hypothesis that rotors preferentially propagate around the slow conducting BZs of patchy fibrosis, which can provide a mechanistic explanation for the improved outcomes of ablation around fibrotic areas.

## Methods

Several authors of this study have recently contributed to the creation of an integrative biophysical model for the entire 3D human atria (Aslanidi et al., 2011; Colman et al., 2013). The model included: (i) detailed atrial electrophysiology based on the Courtemanche-Ramirez-Nattel (Courtemanche et al., 1998) (CRN) atrial myocyte model; (ii) regional heterogeneity in the ionic channel and action potential (AP) properties, and their changes due to AF-induced ionic remodeling, reflected in the respective variations of the CRN model parameters; (iii) atrial geometry provided by the Visible Female anatomical dataset, with the 3D atria segmented into major anatomical regions (Colman et al., 2013). In the current study, the model was further developed to include: (iv) rule-based fiber orientation throughout the entire 3D atria (see in the Supplementary Figure 1; Krueger et al., 2011); (v) a novel model for atrial fibroblast electrophysiology and electrotonic myocyte-fibroblast (M-F) coupling; (vi) patient-specific distributions of fibrosis reconstructed from LGE MRI data and mapped onto the 3D atria model, which accounted for gradual changes of the tissue properties between healthy and fibrotic atrial regions. Detailed description of the existing model components (i–iii), as well as (iv), can be found in previous publications (Aslanidi et al., 2011; Krueger et al., 2011; Colman et al., 2013), and the novel components (v,vi) are described in detail below.

### Atrial fibroblast electrophysiology

Unlike myocytes, fibroblasts are not electrically excitable, however they can maintain a resting membrane potential (RMP), and hence affect and be affected by the tissue electrophysiology. The electrophysiological function of fibroblasts is determined by the presence of a range of ionic channel currents in their cell membrane. It is known that both cultured and freshly isolated fibroblasts express voltage dependant potassium channels (*I*_*Kv*_) (Chilton et al., 2005). A wide variety of currents have now been discovered in the cardiac fibroblast, predominantly potassium (*I*_*Kur*_*, I*_*Kir*_*, I*_*to*_), but also non-specific ionic currents (*I*_*ns*_) which can be mechanically and non-mechanically activated chlorine currents (*I*_*Cl*_). The sources of data for these various currents are diverse, and are summarized in Table 1.

**Table 1:** **Electrophysiological properties of cardiac fibroblasts**.

**Study**	**Fibroblast RMP**	**Currents**	**Cell type**	**Cell source**
Shibukawa et al., 2005	−58 ± 3.9 mV	I_ns_, I_Kv_	Adult rat ventricle	
Chilton et al., 2005	-	I_K1_, I_Kv_	Rat ventricle	Isolated
Kiseleva et al., 1998	−22 mV	-	Rat atria	Isolated
Rook et al., 1992	−20 ÷−30 mV	-	Neonatal rat	Cultured
Kamkin et al., 2003	−37 ± 3 mV	I_ns_^*^	Rat atria	
Wu et al., 2014	−42.8 mV	I_Kur_	Canine atria	Freshly
		I_to_		Isolated
Wang et al., 2003	-	I_ns_^†^, I_BKCa_^‡^	Rat ventricle	Cultured
Miragoli et al., 2006	−14 ÷−25 mV	I_K1_	Neonatal rat ventricle	Cultured
Kohl et al., 2005	-	I_Cl_, I_Kv_, I_K1_, I_Na_	Frog SAN	
Kohl et al., 2005	−15 mV	-	Rat atria and SAN	Isolated and Cultured
Li et al., 2009	-	I_BKCa_, I_to_, I_K1_, I_Cl_^**^	Human ventricle	Cultured

*The table identifies ionic channel currents and RMP of fibroblasts as reported in literature*.

* *(gadolinium sensitive)*,

† *(mechanosensitive)*,

‡ *big conductance Ca^2+^*,

** *(volume sensitive)-activated K^+^ current has been reported in some studies of cultured cells*.

The general approach to modeling atrial fibroblast electrophysiology was based on a well-established ventricular fibroblast model by MacCannell et al. (2007). However, a set of ionic channel currents specific to atrial fibroblasts was considered, based on the CRN formulation of the currents for human atrial myocytes and experimental data from atrial fibroblasts. Data for the atrial fibroblast model was obtained from a study (Wu et al., 2014) on ionic currents in freshly isolated canine atrial fibroblasts. An optimal fit between the modeled ionic currents and experimental data was achieved by varying the current conductances (see Results below). Note that the dog has been widely used as an experimental model for AF (Schotten et al., 2011), and in the absence of ionic current data from human fibroblasts it is sensible to propose that the canine data provides the closest match.

The 4-aminopyridine (4-AP) sensitive current was modeled as *I*_*to, f*_, which is selectively blocked by 4-AP. Fitting the current-voltage (I–V) relationship for this current to experimental data by Wu et al. (2014) involved a 90% reduction in the current conductance compared to its original formulation in the CRN model, but no changes to the CRN current kinetics was introduced.

The S9941 sensitive current was modeled as *I*_*Kur, f*_, as S9941 is a blocker of ionic channels containing the subunit Kv1.5, which forms the channel conducting this current. Fitting the I-V relationship for this current to experimental data by Wu et al. (2014) involved a 40% reduction in the current conductance compared to its original formulation in the CRN model.

The end pulse current was measured by Wu et al. (2014) as the total voltage sensitive current. We considered the remainder of this current after subtracting both *I*_*to, f*_ and *I*_*Kur, f*_ as a sum of a further two currents: a linear non-specific ionic channel current, *I*_*ns, f*_, and the inward-rectifier potassium channel current, *I*_*K*1, *f*_ (see Results below). Both *I*_*Kur, f*_ and *I*_*ns, f*_ are major contributors to the fibroblast RMP. The separation of these two currents ensured that *I*_*K*1, *f*_ reversed at the equilibrium Nernst potential for potassium ions, *E*_*K*_, as is observed in electrophysiological recordings of this current in multiple cell types. The voltage and time dependences for these currents are based on the CRN formulation (index _*f*_ denotes their relevance to fibroblasts):

(1) $$\begin{array}{c} I_{K1,\;f}=\frac{0.03\left(V_{f}+86.75\right)}{\left(1+exp\;\left(0.05\;\left(V_{f}+20\right)\right)\right)} \end{array}$$

(2) $$\begin{array}{c} I_{ns,\;f}=0.018\;V_{f} \end{array}$$

(3) $$\begin{array}{c} I_{to,\;f}=g_{to,\;f}\;*\;{o_{a,\;f}}^{3}\;*\;o_{i,\;f}\;*\;\left(V_{f}-E_{K}\right); \end{array}$$

(4) $$\begin{array}{c} I_{Kur,\;f}=g_{Kur,\;f}\;*\;{u_{a,\;f}}^{3}\;*\;u_{i,\;f}\;*\;\left(V_{f}-E_{K}\right); \end{array}$$

Here, *V*_*f*_ is the membrane voltage of fibroblasts, where *o*_*a, f*,_ *u*_*a, f*_ and *o*_*i, f*,_ *u*_*i, f*_ are activation and inactivation gates, respectively, and *g*_*Kur*_ and *g*_*to*_ are conductances of the respective channels.

The other two currents in the developed atrial fibroblast model are based on the MacCannell et al. (2007) formulation. Specifically, there are currents not measured by Wu et al. (2014) but assumed present in the atrial fibroblast based on experimental studies with other cardiac cells. These are the sodium-potassium exchange current (*I*_*NaK*_) and the background sodium current (*I*_*bNa*_).

Note that in the MacCannell et al. (2007) model and its derivatives, only *I*_*K*1_ and the time and voltage dependant potassium current *I*_*Kv*_ are included. The latter current was shown to be sensitive to 4-AP and S9941 in their experimental work, (Shibukawa et al., 2005) and hence is likely to present a sum of *I*_*to, f*_ and *I*_*Kur, f*_. However, unlike our novel model, MacCannell et al. (2007) modeled it as a single current.

M-F electrotonic coupling mechanism is based on the presence of M-F gap junctions (Rook et al., 1992; Gaudesius et al., 2003; Camelliti et al., 2004), in the same manner as inter-myocyte coupling. In order to simulate the M-F coupling, we used a similar formalism to that developed by MacCannell et al. (2007). Specifically, the CRN atrial myocyte (with the total membrane ionic current *I*_*tot*_) was coupled with the novel atrial fibroblast (with a total membrane ionic current *I*_*tot, f*_) via a linear M-F gap junctional conductance (*G*_*gap*_):

(5) $$\begin{array}{l} I_{tot,\;f}=\sum I_{ion,\;f}=\;I_{to,\;f}+I_{K1,\;f}+I_{Kur,\;f}+I_{ns,\;f}\\ +\;I_{bNa,\;f}+I_{NaK,\;f} \end{array}$$

(6) $$\begin{array}{c} \frac{dV_{f}}{dt}=-\frac{1}{C_{mf}}\left[I_{tot,\;f}\;\left(V_{f},t\right)+G_{gap}\;\left(V_{f}-V_{m}\right)\right] \end{array}$$

(7) $$\begin{array}{c} \frac{dV_{m}}{dt}=-\frac{1}{C_{m}}\left[I_{tot}\;\left(V_{m},t\right)+\overset{n}{\underset{i=1}{\sum }}G_{gap}\;\left(V_{m}-V_{f}\right)\right] \end{array}$$

Here *C*_*m*_ is the membrane capacitance, subscripts *m* and *f* relate to a myocyte and fibroblast, and *n* to the number of fibroblasts coupled to one myocyte. *G*_*gap*_ was set as 0.5 nS (Jacquemet and Henriquez, 2008) and *n* was varied between 0 and 6. Unless specified otherwise, a myocyte was coupled to 2 fibroblasts.

### Patient-specific fibrosis imaging

**Figure 1:**
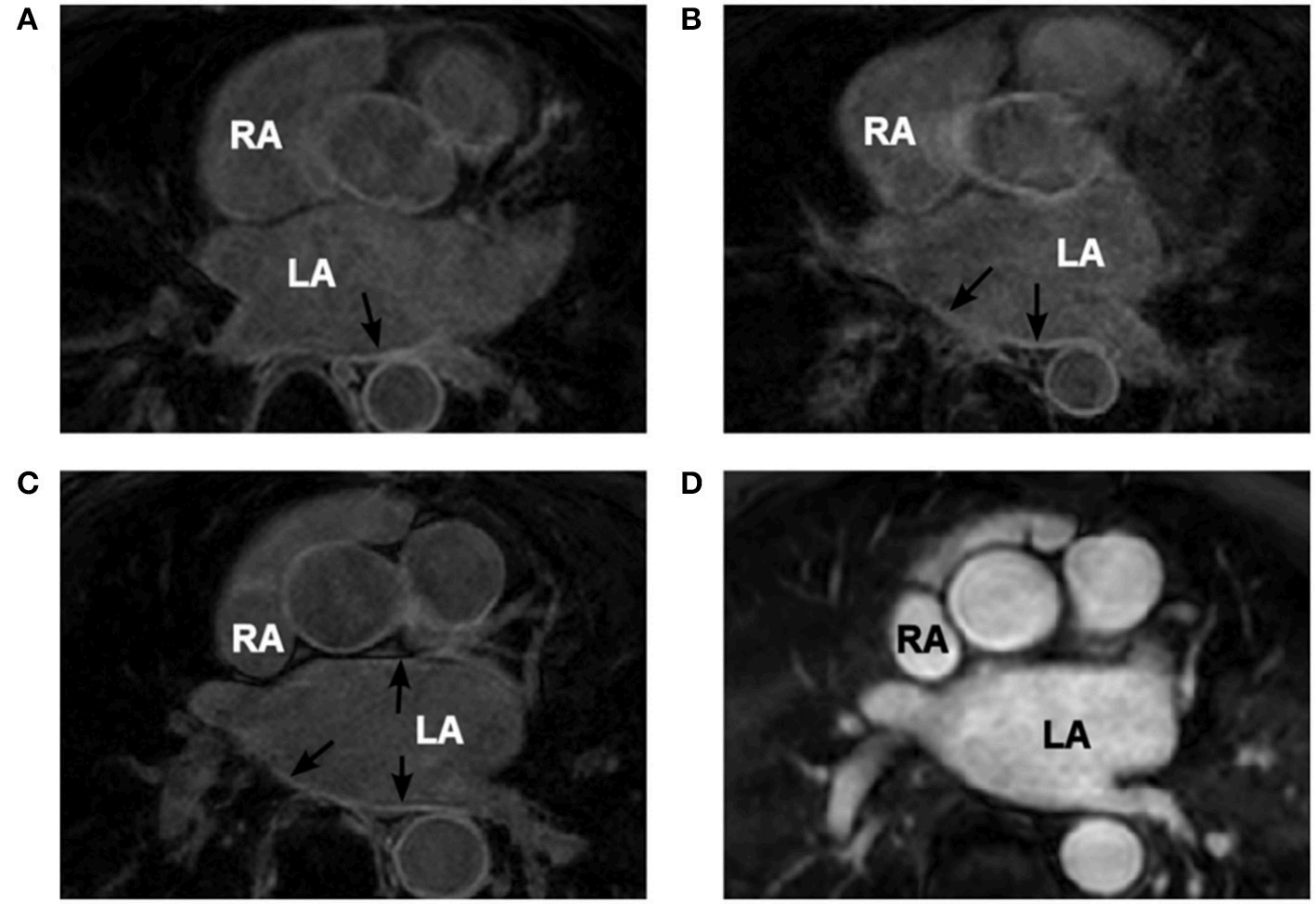
**Patient-specific MRI data. (A–C)** Coronal LGE MRI slices through the atria (two atria are labelled in white) with areas of high intensity linked to fibrosis indicated by the black arrows. **(D)** Gd angiographic MR image with high-intensity blood volume, which is used in the segmentation of the endocardial surface. Images in panels **(C,D)** are from the same patient scan in the same coronal slice.

The images with distributions of atrial fibrosis used in this study were obtained from 3 patients suffering persistent AF and recommended for routine, first time pre-ablation imaging. These data were randomized to ensure patient privacy. A Philips 1.5T MRI scanner was used, and the images were obtained at 1.4 × 1.4 × 4 mm^3^ resolution and saved in DICOM format. Two MRI modalities were used: LGE MRI (Figures 1A–C) and early gadolinium (Gd) angiographic imaging (gated MRA) (Figure 1D). The angiography images were utilized for an improved segmentation of LGE MRI data (see details below). These were taken in the same cardiac phase (atrial diastole) with respiratory gating.

A novel image processing pipeline was developed to reconstruct and register the LGE MRI data on the fibrosis distributions with the 3D atria. The pipeline utilizes fibrosis segmentation tools developed by the CARMA group at the University of Utah, such as plugins to 3D Slicer™. This enables the reconstruction of gradual fibrosis changes as a function of image intensity.

The extraction of fibrosis data from MR images requires thresholding of the intensity of the atrial wall. Obtaining the atrial wall in MRI data is non-trivial (Varela et al., 2015) and detailed segmentation can only be performed manually due to the relatively low resolution and signal to noise ratio (SNR). This process is extremely time consuming and may provide discrepancies between segmenters. However, the LGE MR images are acquired in the same session and at the same cardiac phase as a gated MRA acquisition, used as a navigation image for planning ablation procedures. The gated MRA delineates (Figure 1D) the blood pool which has a high intensity due to the contrast agent (Gd) and allows for accurate semi-automatic segmentation of the endocardial surface. This is achieved using a region growing algorithm within ITK-Snap, which utilizes seed points that iteratively dilate. The growth is limited by image intensity gradients. The segmented endocardial surface then is dilated to provide an epicardial surface. In some cases it required manual rectification in highly convoluted areas. The dilation is usually between 3 and 5 mm, which corresponds to the variable atrial wall thickness (Varela et al., 2015). The endocardial volume is subtracted from the dilated epicardial volume creating a 3D wall mask.

Utilising tools developed by the CARMA group and available as a 3D Slicer plugin (http://capulet.med.utah.edu/namic/cmrslicer/), provides two different methods for fibrosis segmentation. The first, named “*automatic scar segmentation*,” uses a K-means clustering algorithm. This produces a binary fibrosis model in the masked region, identifying the densest fibrosis areas. The second method (“*threshold model*”) collects the MR intensity data from the masked image applying no threshold or segmentation. Both methods project the data onto a surface mesh of the endocardium. This 3D image-based atrial mesh is registered to a respective 3D mesh of the Visible Human Female atria using the IRTK-toolkit (https://biomedia.doc.ic.ac.uk/software/irtk/) using a non-linear deformation. The registered 3D mesh is projected onto the Visible Human dataset using a “nearest neighbor” algorithm. Fibrosis datasets obtained using the “threshold” tool are segmented by LGE MRI threshold, with the threshold values defined on the range of the image intensity in the masked image.

**Figure 2:**
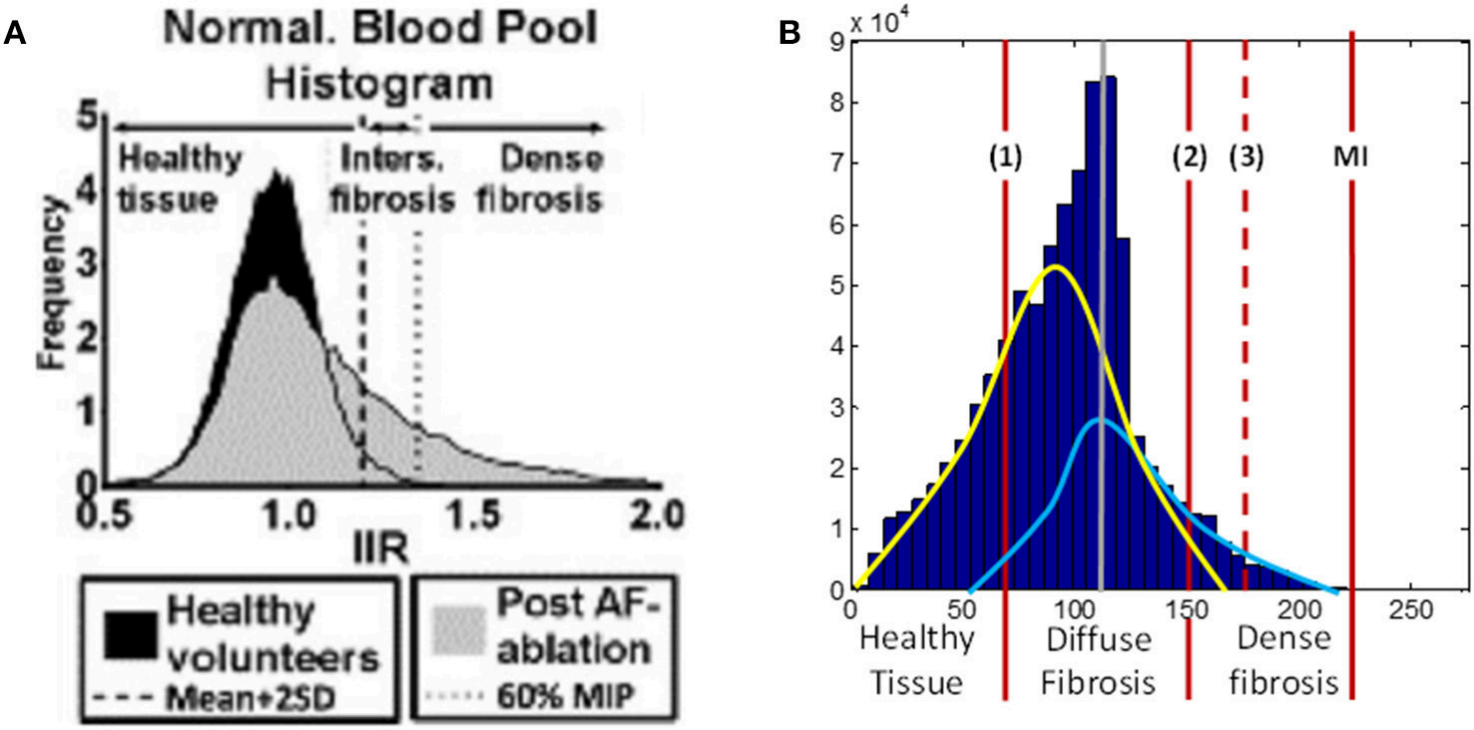
**LGE MRI intensity histograms. (A)** Comparison between healthy volunteers (black shading) and fibrotic (gray shading) tissue, from Benito et al. (2015). Note that this includes the blood pool, with the intensity values normalized by the intensity of blood (hence, the peak at 1 corresponds to blood). The histogram clearly shows a tail of intensities associated with fibrosis. **(B)** Masked intensity histogram, i.e., only the intensity of the voxels within the masked atrial wall region. Two Gaussian curves represent approximations of the distributions of healthy (yellow) and fibrotic (light blue) tissue; the histogram peak (gray line) is the superposition maximum of these two distributions. The red lines show a range for fibrosis level segmentation. (1) is the lower limit of the diffuse fibrosis (levels 1–3) and (2) shows the lower limit for the dense fibrosis regions (levels 4, 5). The dashed red line (3) shows the lower limit of the densest (level 5) fibrosis region. The red line denoted MI shows the maximum intensity from which the fibrosis regions were calculated. MIP, maximum intensity projection; IIR, image intensity ratio.

The range by LGE MRI intensity values corresponding to fibrosis were calculated individually for each patient, due to the intensity variation between scans, using the maximum intensity (MI) as the upper limit (Figure 2B). We hypothesized that the histogram intensity (Figure 2B) was a result of an overlap between two intensity distributions corresponding to healthy and fibrotic tissues (McGann et al., 2008). Thus, the right tail of the histogram was associated with fibrosis, as described in previous studies (Figure 2A), and the left tail of the histogram was associated with healthy tissue.

The range of intensity values was divided into 7, with the lowest 2/7th being allocated to healthy tissue and each fibrosis level, 1–5 allocated as a following 1/7th of the range (moving toward higher values). Due to the densest fibrosis (level 5) covering less tissue than has previously been associated with fibrotic scars (Karim et al., 2013), the highest 1/7th of the range was expanded by 20% (moving toward lower values), with all other parts of the range evenly reduced. This provided significant regions of dense fibrosis in agreement with previous reconstructions. The resultant intensity distribution between levels was as follows: healthy tissue, 0 to 0.27 MI; level 1, 0.27–0.4 MI; level 2, 0.4–0.55 MI; level 3, 0.55–0.69 MI; level 4, 0.69–0.82 MI; level 5, 0.82–1.0 MI. The segmented fibrosis regions were considered to be transmurally uniform in the 3D atrial model.

To investigate the role of segmentation variation, we also created two alternative segmentations for each LGE MRI dataset, one with a higher and another with a lower threshold. The higher threshold case in described above, and the lower threshold case was created by shifting the threshold of dense fibrosis (dashed line in Figure 2B) leftward by 10 units of LGE MRI intensity.

### 3D atrial simulations

For the whole atrial simulations, the monodomain equation was solved on the 3D human atrial geometry with zero-flux boundary conditions, using the forward Euler method with spatial and temporal steps of 0.3 mm and 0.005 ms, respectively. The equation is as follows:

(8) $$\begin{array}{l} \frac{\partial V_{m}}{\partial t}=\nabla \cdot \left(D\nabla V_{m}\right)-\frac{I_{tot}}{C_{m}} \end{array}$$

Here ∇ is the gradient operator and *D* is the diffusion coefficient, which can be a tensor when the tissue is anisotropic. The model uses the 3D fiber vector array to calculate the diffusion tensor to each voxel dependant on the orientation of the fiber (Clayton et al., 2011; Colman et al., 2014). This calculation also requires the longitudinal and transverse diffusion coefficients, *D*_*L*_ and *D*_*T*_, where *D*_*L*_ was constant at 0.3 mm^2^/ms and *D*_*T*_ was varied to change the anisotropy ratio, *R* = *D*_*L*_ /*D*_*T*_, as described below.

The model settings were varied to simulate different conditions linked to fibrosis and AF. Four types of AF-associated remodeling were considered: (1) Ionic remodeling (Colman et al., 2013, 2014), but no structural remodeling (the standard values of *D*_*L*_ = 0.3 mm^2^/ms and *R* = 10:1), which is associated with early-stage AF. (2) Diffuse fibrosis, where instead of ionic remodeling the M-F coupling was added and inter-myocyte coupling was decreased (*D*_*L*_ = 0.12 mm^2^/ms, *R* = 10:1) throughout the atria, both changes associated with short-term AF (Rook et al., 1992; Li et al., 1999). (3) Interstitial fibrosis, where in addition to the presence of diffuse fibrosis atrial tissue anisotropy was increased (*D*_*L*_ = 0.12 mm^2^/ms, *R* = 16:1), which is associated with high level of collagenous depositions in long-term AF (Kawara et al., 2001; Krul et al., 2015). (4) Patient-specific patchy fibrosis (*D*_*L*_ ≤ 0.3 mm^2^/ms, *R* = 10:1) introduced as described below.

The segmented distinctive fibrosis levels, indexed 0–5 depending on the LGE MRI intensity, were applied to simulate the dense-to-diffuse fibrosis gradients in the 3D atrial model. Index 0 represents no fibrosis, and regions with indices 1–5 are modeled as increasingly severe fibrosis. Specifically, indices 0–5 corresponded to progressively increasing M-F coupling (0–5 fibroblasts per myocyte) and decreasing diffusive coupling between myocytes (100–20% of the standard *D*_*L*_ = 0.3 mm^2^/ms). Thus, 3 patient-specific fibrosis distributions were generated.

The tissue effective refractory period (ERP) was calculated in a quasi-1D tissue by applying a stimulating current at one end to initiate AP propagation to the other end. This was modeled as a long 3D slab of 50 × 7 × 7 cells with a space step of 0.3 mm and a diffusion coefficient of 0.3 mm^2^ ms^−1^. The stimulus of −2500 mA was applied in the first 10 layers of cells. This resulted in a 1D plane wave propagating along the slab. Stimuli were applied at a given BCL, followed by a last stimulus applied at a variable S2 interval. The latter was increased until an AP was observed at the non-stimulated end of the cable. The minimum S2 interval for which AP was still able to propagate through the cable was taken as ERP. These simulations also enabled the calculation of the conduction velocity (CV) by measuring conduction times along the slab.

Re-entry was initiated in the 3D model through fast pacing in the RAA or LAA, allowing for the wave to propagate transversely to the crista terminalis (CT) and generate a conduction block.

## Results

### Atrial fibroblast and M-F coupling models

**Figure 3:**
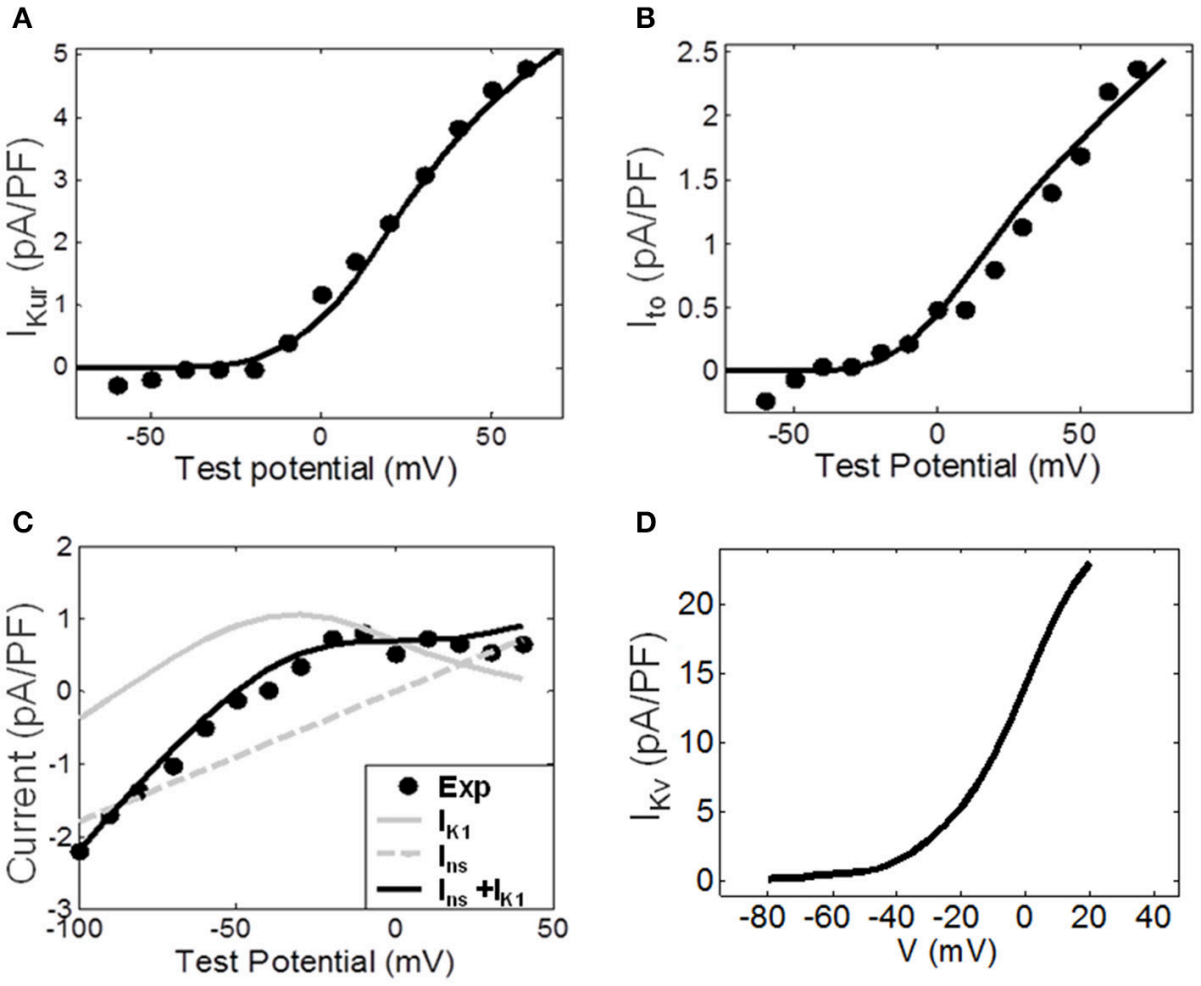
**Ionic channel currents of the atrial fibroblast model. (A–C)** The simulated I-V curves for the major ionic currents (solid lines) are shown along with the respective experimental data (dots). **(A)** The ultra-rapid current, I_Kur_. **(B)** The transient outward current, I_to_. **(C)** The experimentally measured end-pulse current (black dots), with the simulated inward-rectifier and non-specific currents, I_K1_ and I_ns_, (gray lines) and the combined current I_K1_ + I_ns_ (black line). **(D)** I_Kv_ simulated by the ventricular fibroblast model by MacCannell et al. (2007); it represent the sum of I_Kur_ and I_to_. The index ‘f’ used in equations (1)–(4) above is omitted here for simplicity.

The fitting of ionic channel currents in the atrial fibroblast models to the respective patch-clamp data (Wu et al., 2014) can be seen in Figures 3A–D. I-V curves for *I*_*Kur*_, *I*_*to*_ and *I*_*K*1_ (Figures 3A–C) in the model all are in good agreement with the experimental data. After fitting the ionic currents to experimental data, the RMP in the resulting model for a single atrial fibroblast was −42.5 mV, which was in excellent agreement with the respective experimental value of −42.8 mV recorded from atrial fibroblasts (Wu et al., 2014). This value is more positive than the RMP of −48 mV in MacCannell et al. (2007) ventricular fibroblast model, although both are within the experimental data range (Table 1).

**Figure 4:**
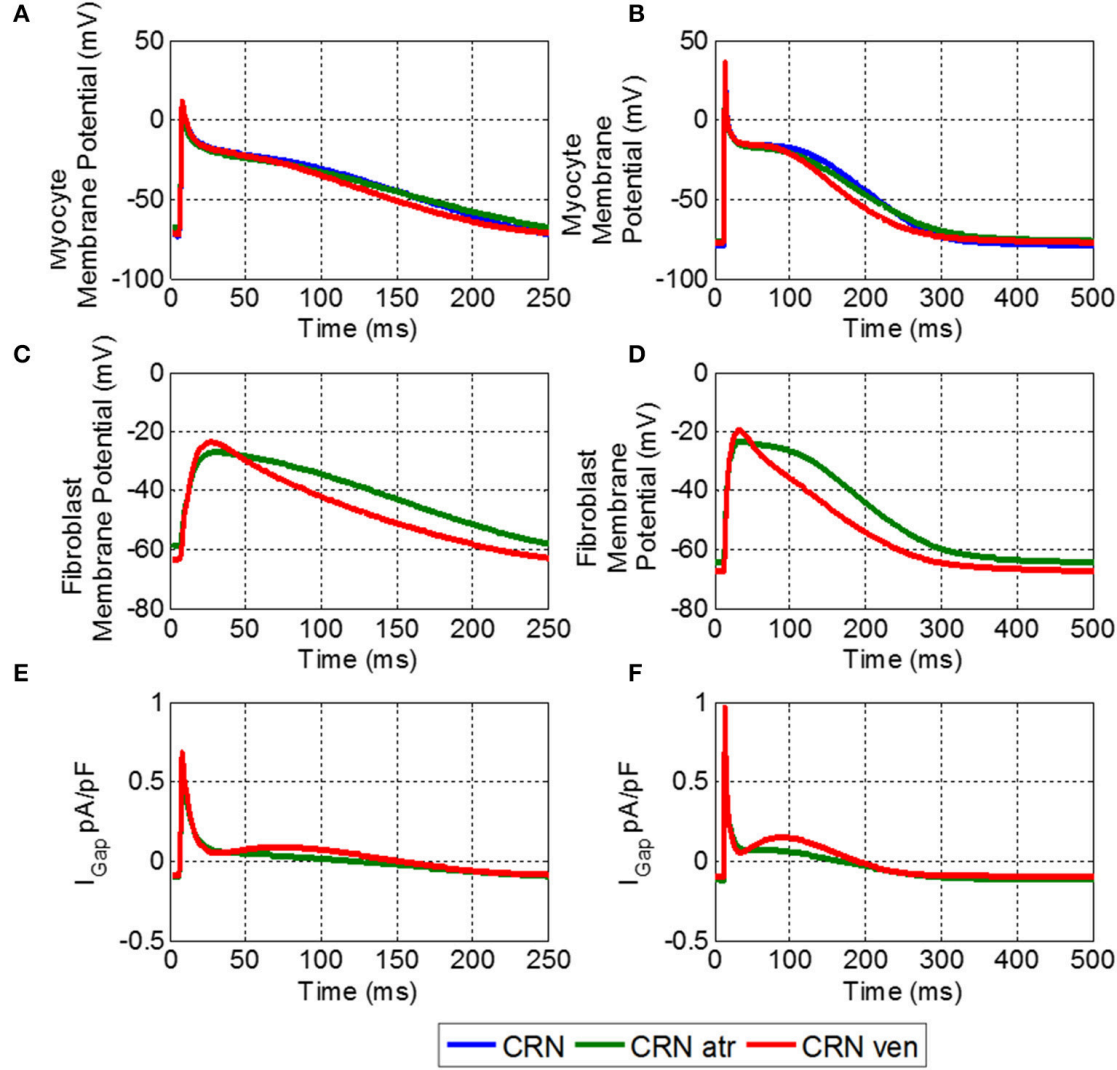
**Effects of M-F coupling on AP in the atrial myocyte described by the CRN model. (A)** And **(B)** show the AP in atrial myocyte in various conditions: uncoupled CRN (blue), coupled with the human atrial fibroblast model (green) and coupled with the ventricular fibroblast model (red). **(C,D)** Show the fibroblast membrane potential for the coupled models, and **(E,F)** show the M-F gap junctional coupling current (IGap). The left hand column **(A,C, and E)** shows these conditions at a BCL of 250 ms and the right **(B,D, and F)** for a BCL of 500 ms.

Coupling to fibroblasts significantly affected electrophysiological properties of the myocyte. Figures 4A,B, illustrates APs in the control CRN model (i) coupled to the developed atrial fibroblast model (ii), and the CRN model for a myocyte coupled to the MacCannell et al. (2007) ventricular fibroblast model (iii). In these simulations, a myocyte was coupled to 2 fibroblasts. The M-F model (ii) produced prolonged repolarisation compared to the uncoupled control model (i), while the M-F ventricular model (iii) produces a more rapid repolarization than in control. Hence, coupling to atrial (ii) and ventricular (iii) fibroblasts produces opposite effects on AP in the CRN atrial myocyte model (i). Figures 4C,D show the respective membrane potential in atrial and ventricular fibroblasts for the M-F coupling models (ii) and (iii).

The fibroblast potentials were purely electrotonic due to the coupling with the myocyte and had substantially lower amplitude than that of the myocyte. The atrial fibroblast had a slower repolarization and slightly lower amplitude than that of the coupled ventricular fibroblast. More importantly, it had a higher RMP, and hence a larger effect of the myocyte RMP.

**Figure 5:**
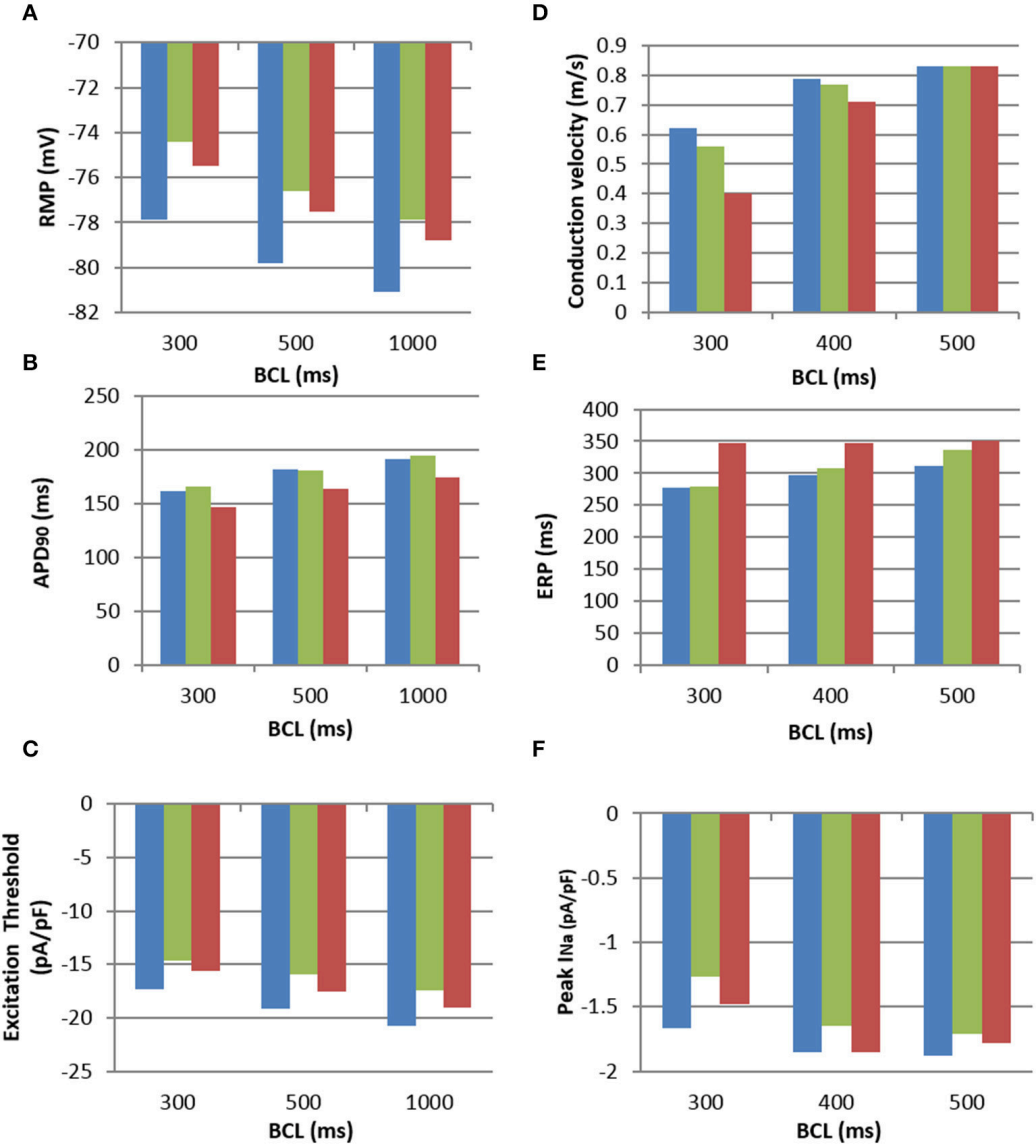
**Electrophysiological characteristics of the M-F coupling models. (A)**, RMP; **(B)**, APD; **(C)**, Excitation threshold in respect to the stimulating current; **(D)**, CV; **(E)**, ERP; **(F)**, Peak sodium current, I_*Na*_, all shown for increasing BCLs. All panels use the same colour code: uncoupled CRN myocyte model (blue), M-F coupling model with atrial fibroblasts (green) and M-F coupling model with ventricular fibroblasts (red).

Figure 5 compares electrophysiological characteristics of the cases (i)-(iii). The RMP (Figure 5A) was increased (became more positive) compared to control in both M-F coupling models (ii) and (iii). At a BCL of 500 ms, in the M-F model (ii) with atrial fibroblast the RMP was −76.5 mV (Figure 5A) and in the M-F model (iii) with ventricular fibroblast the RMP was −77.5 mV, compared to −79.8 mV in the uncoupled CRN model (i). RMP is lowest in case (ii) throughout all BCLs, which can be particularly important at fast rates associated with AF.

The dynamic effects of coupling a CRN myocyte with the fibroblast models significantly changed the refractoriness of the myocyte. There was little difference in APD between the M-F coupling model with atrial fibroblasts (ii) and the control (i) except for at a high pacing rate, e.g., at a BCL of 300 ms (Figure 5B). In comparison, the M-F coupling model with ventricular fibroblasts (iii) reduced the APD throughout the range of BCLs, with a maximum reduction of about 20 ms. Effects of M-F coupling on APD became more pronounced in both models (ii) and (iii) as the number of fibroblasts coupled to a myocyte was increased from 2 to 4 and 6.

The excitation threshold for the stimulus current was also investigated (Figure 5C) for the models. In the M-F coupling model (ii) with atrial fibroblasts, the threshold was −15.9 pA/pF at the BCL of 500 ms. It was significantly lower when compared to both the uncoupled myocyte model (i) (−19.2 mA/mS) and the M-F coupling model (iii) with ventricular fibroblasts (−17.5 mA/mS). Changes of the excitation threshold in models (ii) and (iii) compared to control CRN (i) correlated with the respective changes in the RMP: higher (more positive, but lower absolute value) RMP corresponded to the lower threshold (see Figures 5A,C).

Effects of M-F coupling on ERP were larger than those on APD in both M-F coupling models (ii) and (iii). ERP in the M-F coupling model with atrial fibroblasts (ii) was larger than that in control (i) by about 5–10 ms at BCLs above 300 ms (Figure 5D), but dropped off rapidly at BCL was decreased below 275 ms. ERP in the M-F coupling model with ventricular fibroblasts (iii) was substantially larger than that in control (i) by about 50–100 ms across the range of BCLs.

Despite decreasing the excitation threshold, both M-F coupling models (ii) and (iii) reduced the CV compared to the control (i), with the ventricular M-F model producing the greater reduction. This can be explained by a substantial reduction of the sodium current, *I*_*Na*_, in case (ii) (Figure 5F) and by a large increase of the ERP in case (iii) (Figure 5D). The decrease of *I*_*Na*_ in the myocyte, particularly pronounced due to coupling with atrial fibroblasts at high rates (Figure 5F), can be explained by more positive RMP in atrial fibroblasts (Figure 5A). The latter results in a stronger electrotonic M-F current, *I*_*Gap*_, (Figure 4) and partial inactivation of *I*_*Na*_.

Note that RMP in the novel atrial fibroblast model (−42 mV) was more representative of atrial physiology (Table 1) than that the MacCannell et al. (2007) ventricular fibroblast model (−47 mV), as it was developed based on more comprehensive patch-clamp data from atrial fibroblasts. Moreover, M-F coupling with the ventricular fibroblast model produced non-physiologically high ERP (about 350 ms) and low CV (0.4 m/s) in atrial tissue at high rates (BCL of 300 ms). The high ERP in this case may be a result of slow repolarisation processes, characterized by long time constants, in the ventricular models–whereas our novel atrial fibroblast model uses formalisms, including kinetics inherited from the CRN model, specific to faster repolarisation in the atria. Hence, using the ventricular fibroblast model instead of the atrial fibroblast model in 3D human atrial models could lead to different behavior in AF, as shown in the next section.

### Uniform fibrosis in 3D atrial models

**Figure 6:**
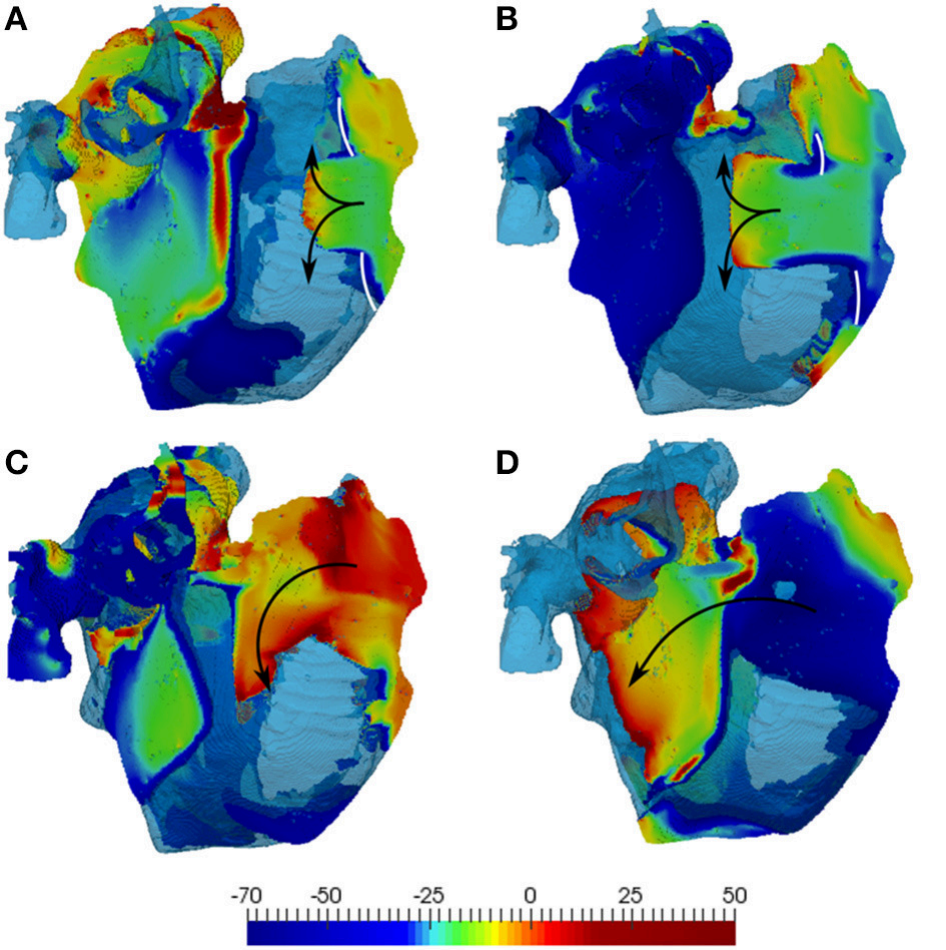
**Simulations of the 3D human atrial model with diffuse fibrosis**. The latter includes normal 10:1 anisotropy, 60% reduction of diffusion coefficients and M-F coupling with 2 fibroblasts. **(A)** And **(C)** illustrate simulations with the developed atrial fibroblast model, and **(B,D)** show those with the MacCannell et al. (2007) ventricular fibroblast model. The top panels **(A,B)** shows the initial conduction blocks (white lines), and **(C,D)** show re-entry generation. 3D voltage maps (rainbow palette) are superimposed onto the atrial geometry (transparent blue). Wave directions in the RA are indicated by black arrows, with secondary waves travelling away into the LA.

In 3D atrial simulations, fast pacing in the anterior RA resulted in a conduction block leading to wave breaks and re-entry in all simulated conditions (see Methods). The precise location and timing of the wave-block varied depending on the condition considered and pacing rate used, but was always clearly observed between the CT and the pacing site (seen in Figure 6).

In the diffuse fibrosis conditions, M-F coupling with atrial and ventricular fibroblasts resulted in greatly varying duration of re-entry: about 3 and 12 s, respectively. A much longer duration of re-entry in the latter case can be explained to the wavelength reduction associated with extremely low CV values seen with this model (Figure 5D) at high rates. The short wavelength can be seen in Figure 6 as a larger excitable gap (between a re-entering wave and the preceding one) in the case of coupling with ventricular fibroblasts (Figures 6A,C), as compared to that with atrial fibroblasts (Figures 6B,D). This also points the sensitivity of 3D atrial behavior to the choice of a fibroblast model, especially at high rates typical of AF.

**Figure 7:**
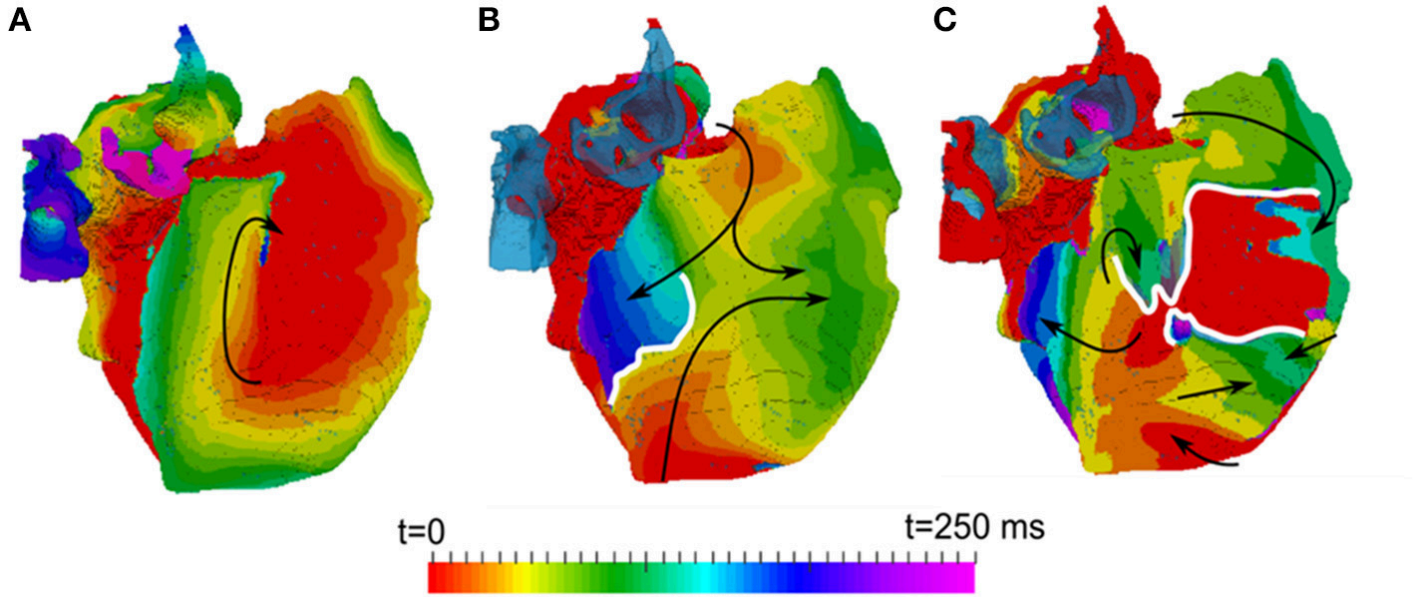
**Activation maps in the 3D atria model for typical patterns of observed re-entrant behaviours. (A)** A stable rotor, seen in the conditions with ionic remodelling. **(B)** Unstable re-entrant waves in the conditions with diffuse fibrosis. **(C)** wave breakdown in the conditions with interstitial fibrosis. Wave propagation directions are shown by black arrows and wave-blocks are shown by white lines. In **(B,C)**, the left atrium is fully activated at the initial moment of time *t* = 0 and the PVs are repolarized and not activated (and hence, seen in the transparent blue color corresponding to the tissue geometry) within the range of these activation times.

In all diffuse fibrosis conditions, re-entry was unstable and self-terminated after several seconds (Figure 7B). In the ionic remodeling condition, known to lead to a large decrease of APD and the wavelength (Aslanidi et al., 2011; Colman et al., 2013), a single stable rotor persisted through the entire simulation (Figure 7A). Another pattern was observed in the high anisotropy condition linked with interstitial fibrosis. Re-entrant waves initiated due to the wave break at the CT and a relatively short wavelength were afterwards broken down into multiple secondary wavelets (Figure 7C). The breakdown mechanism was due to the presence of a higher number of sites with slow transverse conduction (similar to the CT). The general 3D atrial activation pattern of multiple wavelets propagating quasi-chaotically and independently was typical of AF.

However, stabilization of sustained activity in specific atrial locations was not observed in any of the uniform conditions considered. In the next section, such a stabilization of re-entrant wave is linked with patient-specific areas of heterogeneous patchy fibrosis in the 3D atria.

### Patient-specific fibrosis in 3D atrial models

**Figure 8:**
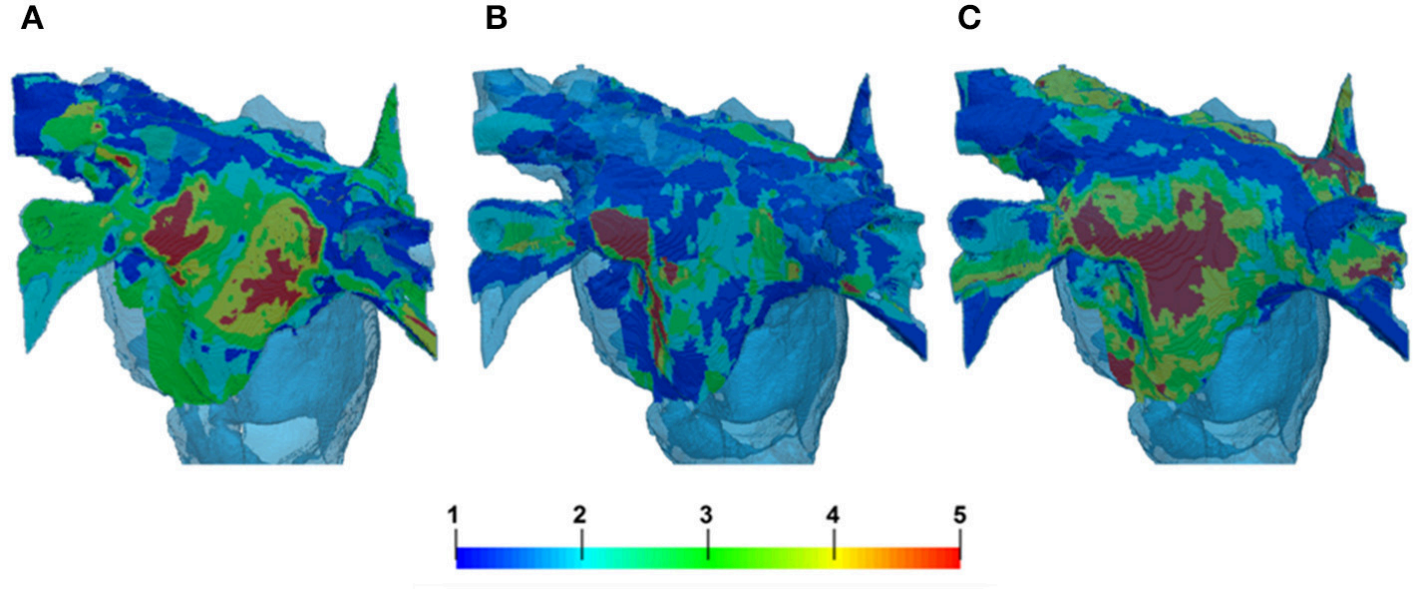
**Reconstruction of fibrosis by LGE MRI intensity threshold method from 3 AF patients**. Variation in distribution can clearly be seen between patients in panels **(A–C)**. **(A,C)** Illustrate cases with lower thresholds and B shows a case with higher threshold. The palette below shows the reconstructed levels of fibrosis, from diffuse (levels 1–2) to patchy (levels 3–4) and dense (level 5). 3D atrial geometry is also shown (transparent blue), similar to Figures 6, 9.

The segmentations of LGE MR images yielded different fibrosis distributions in 3 AF patients. The reconstructed fibrosis distributions varied in the extent and severity, as seen in Figure 8. All datasets showed areas of more severe, dense fibrosis (corresponding to the highest LGE MRI intensity) in the posterior LA (Figure 8), which is a common ablation target. The relative quantized distributions of fibrosis in the thresholded datasets showed a higher distribution in the lower fibrosis levels. Significantly lower amounts of fibrosis were observed in the higher levels (4–5) despite the histogram bias for level 5 fibrosis. In all the datasets good connectivity of fibrotic regions was observed, with the fibrosis levels changing gradually (Figure 8).

**Figure 9:**
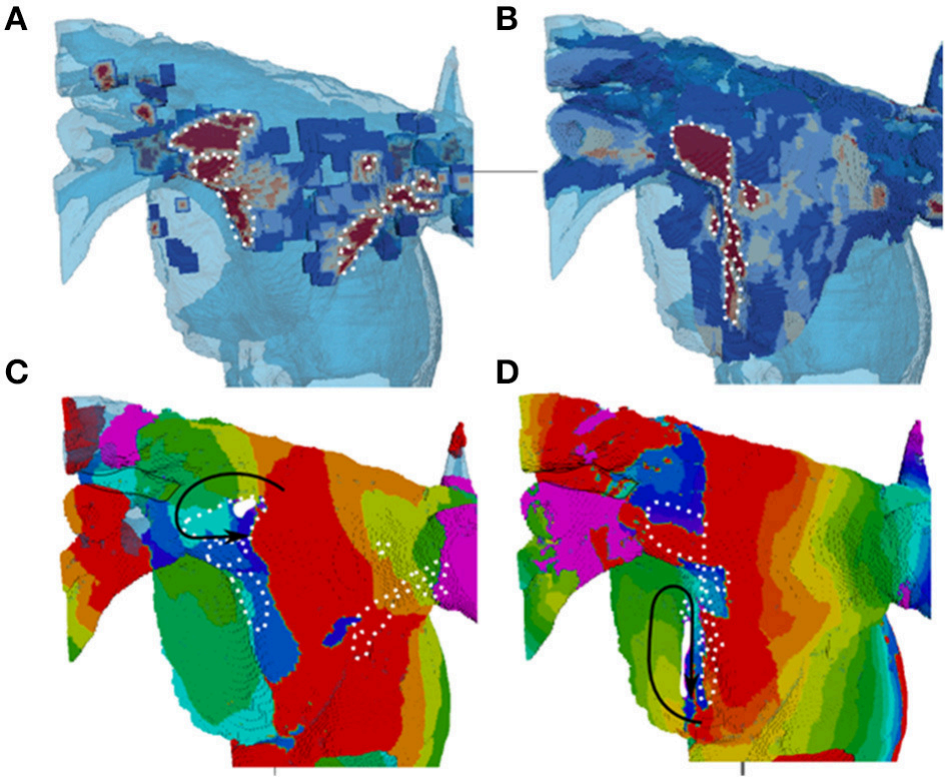
**Stabilization of rotors in patchy fibrosis regions. (A,B)** Show two different patient-specific fibrosis distributions, and **(C,D)** Show the respective single rotor period activation maps (see color code in Figure 7). The maps illustrate the variation of rotor pinning specific to the distributions of fibrosis. Wave propagation direction is denoted by the black arrow, the core of the rotor is indicated by the solid white shape and central dense fibrosis regions are indicated by the dotted white lines. **(C)** Shows a rotor pinned directly to a dense fibrotic region (the dynamics of rotor movement in this case is illustrated by the Supplementary Video 1, and the respective fiber orientation in the 3D atria is shown for comparison in the Supplementary Figure 1). **(D)** Shows a rotor with the core adjacent to a dense fibrotic region, but rotating within a border zone.

In 3D atrial model simulations, variable duration pinning to the fibrotic region was typically seen for all patient-specific cases (Figure 9). Permanent rotor stabilization in the BZ was seen in two cases, where rotor was pinned until the end of simulation. One simulation produced rotor movement directly around an inner area of dense fibrosis (Figure 9A) and another within an adjacent border zone of less severe patchy fibrosis (Figure 9B). In both cases, the rotor core was localized within regions of broadly defined patchy fibrosis (levels 3–4). Spatio-temporal dynamics of the rotors is illustrated in Figures 9C,D and Supplementary Video 1.

**Figure 10:**
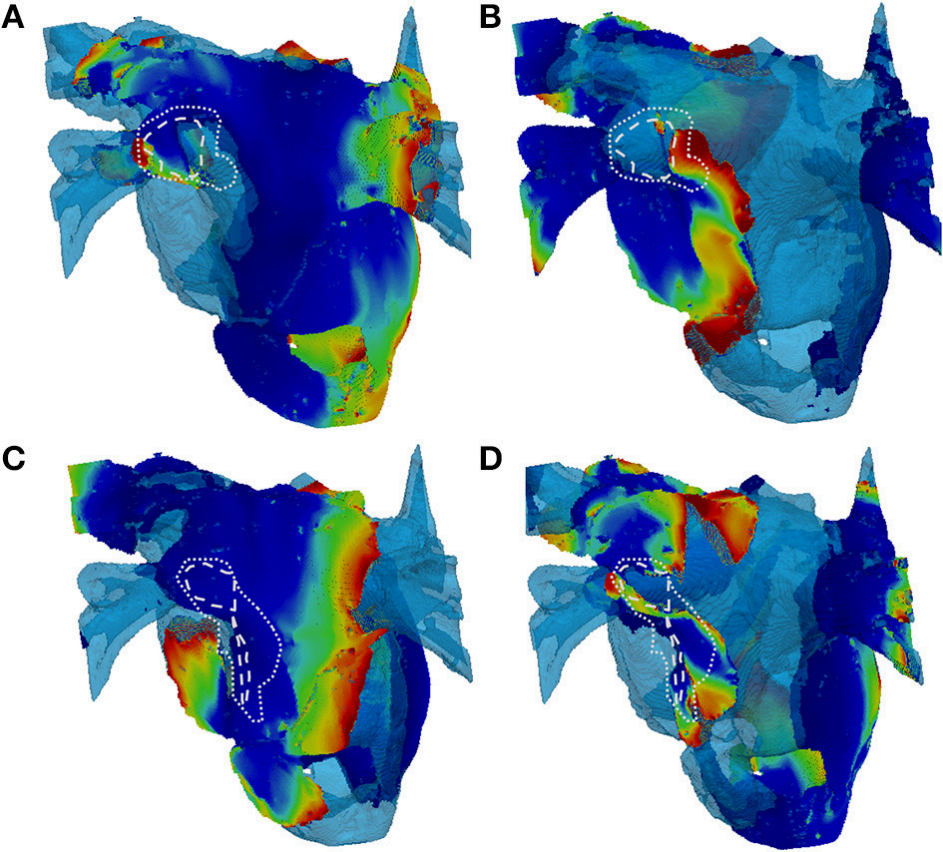
**Snapshots of re-entry in 3D atria with fibrosis distributions based on high and low thresholds of LGE MRI intensity datasets. (A,C)** Show 2 patient cases with a high threshold (lower amount of fibrosis) and **(B,D)** Show the respective cases with a low threshold (higher amount of fibrosis). The rows delineate different patients **(A–D)**. In the high threshold cases **(A,C)**, only a short lived rotor (indicated) was present in the LA. In the high threshold cases **(B,D)**, multiple re-entrant waves are present in the LA fibrotic regions. White dashed and dotted lines indicate level 5 (dense) and levels 3–4 (patchy) levels of fibrosis, respectively. 3D voltage maps are superimposed onto the atrial geometry (see color code in Figure 6).

In patient-specific 3D atrial model simulations with variation of LGE MRI intensity threshold, different behaviors were observed (Figure 10). In simulations with the higher thresholds (lower fibrosis level) no stable rotor pinning was observed, with only short lived (less than 1 s) meandering re-entry in the LA fibrotic regions in two patients (Figures 10A,B). Simulations of the same two patient-specific atrial models with the lower threshold (higher fibrosis level) showed severe wave breakdown, with multiple re-entrant wavelets in the LA and to a lesser extent the RA. Interactions of the wavelets in fibrotic regions prevented the generation of a single rotor (Figures 10B,D). The complex electrical activity in both patient-specific 3D atria was characteristic of AF and sustained for the entire simulation (10 s).

The 3D atrial simulations performed for this section provide further evidence to the importance of using realistic fibrosis patterns to study the dynamics of AF, and elucidate the role of fibrosis in arrhythmogenesis due to the unique wave interactions for different fibrotic distributions.

## Discussion

In this study, cell-to-organ models of the human atria were developed and applied to explore the effects of fibrosis on AF arrhythmogenesis. Fibrosis was modeled by integrating (i) a novel electrophysiologically detailed model for a single atrial fibroblast, (ii) electrotonic myocyte-fibroblast (M-F) coupling, (iii) structural effects of fibrosis on anisotropic conductivity of atrial tissue and (iv) either uniform or patient-specific distributions of fibrosis in the 3D atrial model. Patient-specific distributions were reconstructed from clinical LGE MRI and angiography data, and a novel image processing pipeline was developed to maps the distributions of fibrosis into the 3D atrial model and investigate the effects of regional fibrosis patches on AF.

The modeling revealed that: (1) electrophysiological properties of atrial fibroblasts are different from those of ventricular fibroblasts, with the largest effect of atrial M-F coupling being on the myocyte RMP and *I*_*Na*_, and leading to a reduction of atrial CV; 2) further to the M-F coupling, effects of fibrosis on tissue coupling and anisotropy greatly reduce atrial CV; 3) heterogeneous distributions of patchy fibrosis can result in rotor slowing and stabilization in fibrotic borders. The latter result provides a mechanistic explanation for the observations of recent studies (Jadidi et al., 2013; Haissaguerre et al., 2016; Zahid et al., 2016) linking patient-specific atrial fibrosis distribution and AF arrhythmogenesis.

### Atrial fibroblast model

A novel model for atrial fibroblast electrophysiology was developed based on experimental data by Wu et al. (2014) and applied to study the effects of M-F coupling with the atrial myocyte. Atrial fibroblasts in the model had higher (more positive) RMP compared to the previous models of ventricular fibroblasts by MacCannell et al. (2007) and hence a stronger effect on the excitability of atrial myocytes. This is in agreement with previous studies showing that the RMP of fibroblasts can have significant effects on AP properties of the myocyte (Jacquemet and Henriquez, 2008; Maleckar et al., 2009).

During the early phases of AP, fibroblasts in the M-F model act as a current sink effectively reducing the upstroke of the myocyte, with the AP amplitude (APA) reduced by 5 mV with the atrial fibroblast model (Figure 4). Similar behavior has been shown in other studies (Jacquemet and Henriquez, 2008; Maleckar et al., 2009), but with larger M-F coupling conductances. The APA reduction can slow down the propagation in atrial tissue due to the reduced electrotonic current transferred to neighboring myocytes. During the later phases of the AP, the fibroblasts in the M-F model act as a current source, prolonging the repolarisation phase (Figure 4) and increasing ERP (Figure 5E) in the myocyte.

The latter result is similar to the findings by Jacquemet and Henriquez (2008) and Maleckar et al. (2009) who both used a modified MacCannell et al. (2007) model for ventricular fibroblasts. However, the greatly prolonged ERP in the atrial myocyte coupled to ventricular fibroblasts (Figure 5E) is non-physiological, and also results in non-physiologically large reductions in atrial CV (Figure 5D). Coupling to the novel atrial fibroblast model produced more physiological values of atrial ERP and CV (Figure 5). A relatively small CV decrease in this case also had a different mechanism. The excitation threshold in the myocyte coupled to atrial fibroblasts was decreased (Figure 5C) due to increased RMP in the myocyte (which was caused by inflowing *I*_*Gap*_ from the atrial fibroblasts with relatively higher RMP). Counter-intuitively, the decreased threshold did not result in increased atrial CV–in fact, CV was significantly decreased (Figure 5D). This was due to the inflow of *I*_*Gap*_ into the myocyte leading to a partial inactivation of *I*_*Na*_ (Figure 5F).

Thus, the developed model for atrial fibroblast is more relevant to atrial electrophysiology (Table 1) than the existing ventricular fibroblast models, and also provides more relevant insights into the arrhythmogenic effects of M-F coupling in the atria. The latter include CV reductions that can be responsible for regional conduction blocks and the generation of re-entrant waves. The fibroblast model provides a valuable tool for further studying AF in 3D atrial models. The model could be further validated if novel data from human atrial fibroblasts become available.

### 3D effects of uniform fibrosis

The 3D atria modeling enables exploring the interplay between effects of fibrosis on atrial structure and function and the resulting AF scenarios. Three uniform effects of AF-induced remodeling in the entire 3D atrial model were considered: ionic remodeling, diffuse fibrosis (M-F coupling + decreased M-M coupling) and interstitial fibrosis (diffuse fibrosis + increased atrial tissue anisotropy). Simulations showed a range of re-entrant behaviors (Figure 7).

Similar to previous studies (Colman et al., 2013), the 3D model was first validated against atrial activation data in sinus rhythm, demonstrating a good agreement with clinical measurements by Lemery et al. (2007) Further simulations of ionic remodeling effects also agreed with previous results (Aslanidi et al., 2011; Colman et al., 2013), showing a stable rotor in the 3D atrial model in the case when M-M coupling was normal (Figure 7A). This was due to the remodeling greatly decreasing atrial APD, and therefore the wavelength.

In case of diffuse fibrosis, simulations produced unstable re-entry that self-terminated after several seconds. This was due to the fact that APD and ERP in this case was relatively high (Figures 5A,E, 7B), which resulted in a long wavelength comparable to the size of the atria. Re-entry was sustained longer in the model with coupling to ventricular fibroblasts compared to atrial fibroblasts (12 vs. 3 s), due to low CV (Figure 5D), and hence the wavelength (Figure 6), in the former case. These results also point to the sensitivity of 3D atrial behavior to the choice of a fibroblast model, which should be relevant to atrial electrophysiology.

In case of interstitial fibrosis, AF-induced increase in tissue anisotropy (Kawara et al., 2001; Koura et al., 2002; Verheule et al., 2013; Angel et al., 2015; Krul et al., 2015) resulted in multiple conduction blocks and wave breakdown throughout the 3D atria (Figure 7C). This was the only condition where the 3D atria model produced a sustained AF-like patterns with multiple re-entrant wavelets. Overall, changes of the 3D wave dynamics due to the increased anisotropy were more significant than the effects of M-F coupling or wavelength reduction due to ionic remodeling (Figure 7). This suggests that the structural effects of fibrosis, which develop in later stages in AF progression, may play the most important role in AF sustenance. However, stabilization of sustained activity in specific atrial locations (Haissaguerre et al., 2016), was not observed in any of the uniform cases, and can only be seen with heterogeneous distributions of fibrosis.

### Effects of patchy fibrosis in 3D atria

The 3D atrial simulations demonstrated unique wave interactions with different fibrotic distributions, and hence the importance of using patient-specific LGE MRI data to study AF.

The reconstructed LGE MRI patient datasets showed increased fibrosis distribution on the LA posterior wall (Figure 8). This region may be statistically prone to fibrotic infiltration and is often a site where complex fractionated electrograms are identified, which are common targets for ablation. The lower extent of fibrosis on the LA roof and floor may be due to the lower Z axis resolution of the MRI scan. Regardless of limitations of the imaging method, LGE MRI intensity has been strongly linked to fibrosis, and the reconstructed fibrosis distributions agree with existing knowledge from various modalities.

In 3D atrial simulations with patient-specific fibrosis distribution, a clear stabilization of rotors in regions of patchy fibrosis was observed in 2 out of 3 cases. One case produced a larger circus movement around a dense fibrosis region (Figure 9C) and another produced a smaller extent movement along the border of a dense region (Figure 9D). In both cases, rotors moved in slow-conducting BZ of patchy fibrosis surrounding inner regions of dense fibrosis. Note that fiber orientation can also be important in determining the rotor dynamics (Colman et al., 2014). However, in our simulations slow conduction in the fibrotic BZ appeared to be the main determinant of the rotor location–without patchy fibrosis, rotors meandered freely as seen in Figures 6, 7.

Further simulations also showed that patient-specific fibrosis distributions can induce wave breakdown (Figure 10) which is symptomatic of AF. This was only observed in simulation of high levels of fibrosis (obtained with a low LGE MRI intensity threshold) and resulted in permanent AF for the duration of the simulation (10 s). Smaller re-entrant wavelets were observed in these simulations moving chaotically throughout the fibrotic region. Contrarily, simulations of lower levels of fibrosis in the same patient (obtained by using a higher LGE MRI intensity threshold) produced very short duration re-entry that was neither stable nor resulted in wave breakdown. Note that the overall amount of fibrosis was not very different between the high and low thresholds. Rather, fibrosis was redistributed toward a higher proportion of severe fibrosis (levels 3–5) with an overall longer slow-conducting BZ. Hence, the rotor dynamics in AF may be determined primarily by the presence of such distributions.

Our results are in agreement with previous computational studies of atrial fibrosis: McDowell et al. (2015) and later Zahid et al. (2016) have shown that rotors in 3D atrial models can be localized near areas of fibrosis introduced based on LGE MRI data. Similar observations were made in cases of discrete fibrotic zones (Zahid et al., 2016), statistical distributions of fibrotic properties from dense fibrosis to healthy tissue (McDowell et al., 2015), and the respective continuous distributions in the current study. Hence, the mechanism of rotor stabilization proposed in our study can be independent on a specific way of modeling fibrosis. The accuracy of modeling fibrosis can be important for finding precise location of the rotors, and our approach of reconstructing continuous areas of patchy fibrosis may provide an advantage over models based on binary (Zahid et al., 2016) or statistical (McDowell et al., 2015) reconstructions.

## Limitations

One of the major limitations of this work is a lack of experimental validation for the electrophysiological effects of fibrosis at the atrial cell and tissue level, such as its effects on APD and ERP. However, the same lack of data highlights the importance of modeling and simulation in order to better understand the problem. Further experimental measurements *in-vivo* are required for the validation of the M-F coupling effects observed in the models. Another is a lack of comprehensive clinical validation at the 3D atrial level, although results of our simulations are in agreement with observations of the existing studies (Jadidi et al., 2013; Haissaguerre et al., 2016; Zahid et al., 2016).

Although LGE MRI and the threshold segmentation methods also require further validation, this study aimed to investigate the effect of substantial fibrosis regions, which can be clearly seen in LGE MR images. The difference between the high and low levels of fibrosis also shows that the developed image processing and modeling pipeline is sensitive to the choice of parameters (such as LGE MRI intensity threshold) and can yield very different results. Whilst the quantification for LGE MRI intensity and amount of fibrosis from patients is currently limited, the LGE MRI intensity should indicate areas of higher fibrosis (McGann et al., 2014) in comparison to the rest of the tissue. The LGE MR images may also suffer from partial volume averaging, in which neighboring tissues influence the wall mask because of the coarse resolution of the image.

## Conclusion

Clinically observed correlation between AF progression and fibrosis levels in the atria have led to suggestions of an empirical link between the two. The aim of this work was to understand and substantiate this link with the aid of mechanistic cell-to-organ atrial modeling. We showed that both structural and functional effects of fibrosis result in reduction of atrial CV, and that rotors sustaining AF typically propagate in the slow conducting BZs of patchy fibrosis. These findings provide a mechanistic explanation for the improved outcomes of ablation around fibrotic areas, which can be based on the elimination of the slow-conducting substrate in the fibrotic BZ (fibrosis levels 3–4), and may help in future patient-specific planning of such procedures.

## Author contributions

All authors have made substantial contributions to this study. RM and OA conceived and designed the study, and drafted the manuscript. RM substantially contributed to data analysis and computer simulations, and OA substantially contributed to the interpretation of the results. MC, HC, and GS contributed to data analysis, computer simulations and manuscripts editing. All authors have also approved the final version to be published while agreeing to be accountable for all aspects of the work in ensuring that questions related to the accuracy or integrity of any part of the work are appropriately investigated and resolved.

## Funding

This work was supported by the British Heart Foundation (PG/15/8/31130).

### Conflict of interest statement

The authors declare that the research was conducted in the absence of any commercial or financial relationships that could be construed as a potential conflict of interest.
