# Supplementary material for: Slow Conduction in the Border Zones of Patchy Fibrosis Stabilizes the Drivers for Atrial Fibrillation: Insights from Multi-Scale Human Atrial Modeling
Source: Front Physiol. 2016 Oct 25;7:474. doi: 10.3389/fphys.2016.00474 (PMC5079097; doi:10.3389/fphys.2016.00474)
Supplement: Supplementary file 2 [file Image1.PDF]

Supplementary Material

Slow conduction in the border zones of patchy fibrosis stabilises the drivers for atrial fibrillation: Insights from multi-scale human atrial modelling

Ross Morgan, Michael A. Colman, Henry Chubb, Gunnar Seemann, Oleg V. Aslanidi*

*** Correspondence:** Corresponding Author: oleg.aslanidi@kcl.ac.uk

1 Supplementary Data

Supplementary Video 1. Spatiotemporal dynamics of electrical activity in the 3D human atria (color code explained in Figure 6 of the manuscript), with an area of patchy fibrosis (grey) in the left atrium.

1.1 Supplementary Figures

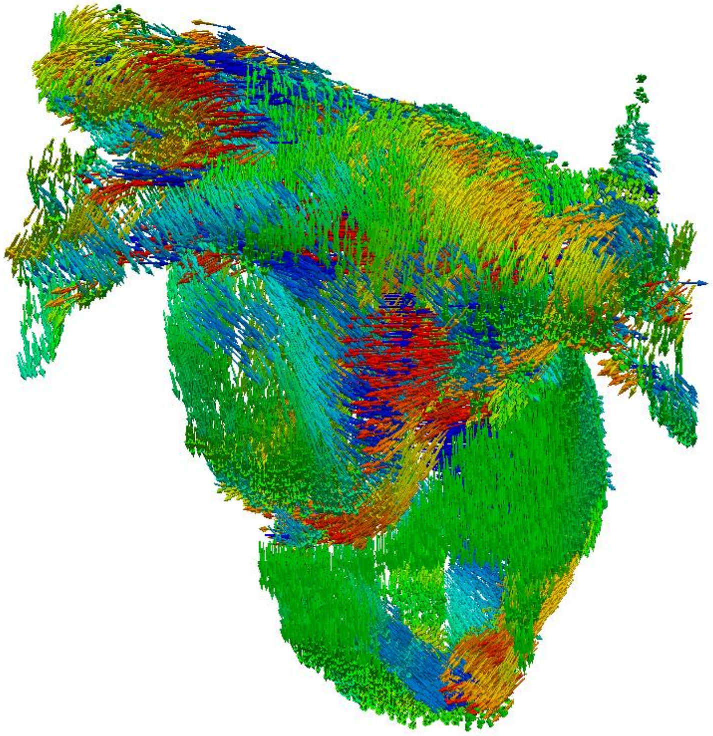

Supplementary Figure 1. Fibre orientation in the 3D human atrial model. Atrial fibres are coloured according to the local fibre orientation component along the anterior-posterior direction.
